# Diseases of the Eye

**Published:** 1855-07

**Authors:** 


*Diseases of the Eye.*

It may be of interest to our readers to know that the managers of the Mercy Hospital in this city, have established a ward in connection with the medical department of the Hospital, specially for the reception and treatment of Diseases of the Eye. The accommodations are very comfortable, and patients can rely on the most careful and well-directed attention.

A pretty full report in reference to the diseases treated in this institution during the past year will be given in our next issue.

